# Alternol-Induced Oxidative Modification of SQSTM1/p62 Is Associated with Nrf2 Signaling and Autophagy-Related Responses in Prostate Cancer Cells

**DOI:** 10.3390/antiox15060779

**Published:** 2026-06-22

**Authors:** Wang Liu, Jiang Zhao, Changlin Li, Haixia Xu, Ruibao Chen, Xing Zeng, Jun Yang, Cuncong Zhong, Xiangwei Wang, Benyi Li

**Affiliations:** 1 Department of Urology, The University of Kansas Medical Center, Kansas City, KS 66160, USA; wliu6@kumc.edu (W.L.); serican.academy@gmail.com (H.X.); rbchen@tjh.tjmu.edu.cn (R.C.); 2013tj0589@hust.edu.cn (X.Z.); jyang0105@hust.edu.cn (J.Y.); 2 Department of Urology, The Affiliated Hospital of Guangdong Medical University, Zhanjiang 524001, China; urologyzhaoj@tmmu.edu.cn; 3 Tianjin Institute of Urology, The Second Hospital of Tianjin Medical University, Tianjin 300211, China; changlinli@mail.nankai.edu.cn; 4 Department of Electrical Engineering and Computer Science, The University of Kansas, Lawrence, KS 66045, USA; cczhong@ku.edu

**Keywords:** p62, *SQSTM1*, autophagy, Nrf2, oxidative stress, p62 protein aggregation

## Abstract

SQSTM1/p62 is a multifunctional scaffold protein that plays important roles in selective autophagy and cellular redox homeostasis. While phosphorylation-dependent regulation of p62 has been extensively studied, the functional significance of oxidative modification remains incompletely understood. Our previous studies showed that the natural small compound Alternol induces cancer cell-specific killing via a xanthine oxidase-mediated strong oxidative stress. In this study, we investigated p62-associated oxidative responses under Alternol-induced oxidative stress conditions in prostate cancer cells. Using biochemical assays and cell-based models, we found that Alternol treatment was associated with the accumulation of oxidized and high-molecular-weight p62 species, accompanied by altered KEAP1 association and increased Nrf2-associated signaling. Furthermore, Alternol-induced p62 oxidative modification was associated with autophagy-related responses and adaptive cellular survival under oxidative stress conditions. Disruption of the Cys105/113-dependent oxidative modification response attenuated Nrf2-associated transcriptional activity and increased cellular sensitivity to Alternol treatment. Collectively, our findings support an association between p62 oxidative modification and redox-responsive autophagy- and antioxidant-associated signaling pathways under Alternol-induced oxidative stress conditions, providing new insight into adaptive stress responses in prostate cancer cells.

## 1. Introduction

Sequestosome 1 (SQSTM1/p62) is a multifunctional scaffold protein involved in multiple cellular processes, including autophagy, protein quality control, oxidative stress response, and signal transduction [1]. As a selective autophagy receptor, p62 participates in the recognition and degradation of ubiquitinated cargo and contributes to the maintenance of cellular proteostasis under stress conditions. Emerging evidence has also implicated p62 as an important regulator of redox signaling pathways involved in cancer cell survival and adaptation [2,3].

Reactive oxygen species (ROS) are important regulators of autophagy and stress-responsive signaling pathways in cancer cells. Excessive ROS accumulation can activate autophagic pathways through multiple mechanisms, including ROS-FOXO3-LC3/BNIP3 and ROS-Nrf2 signaling cascades [2,3,4]. In addition to functioning as signaling molecules, ROS can directly modify proteins through oxidative post-translational modifications, thereby altering protein structure, stability, and activity. Recent studies suggest that oxidative modification of p62 represents an important mechanism linking redox imbalance to autophagy regulation and cellular stress adaptation [5,6]. However, the functional significance of p62 oxidation in response to pharmacologically induced oxidative stress in cancer cells remains incompletely understood.

Alternol is a small natural compound derived from the fermented extracts of a mutant fungus *Alternaria alternate var. monosporus* [7]. Our previous studies demonstrated that Alternol elicited a significant ROS accumulation and triggered apoptotic cell death preferentially in prostate cancer cells while leaving benign prostate epithelial cells [7,8]. We further showed that Alternol-induced oxidative stress contributes to endoplasmic reticulum stress and immunogenic cell death responses in prostate cancer models [9,10]. Although ROS generation appears to be a major mediator of Alternol-induced cellular responses, the downstream adaptive mechanisms triggered by oxidative stress remain largely unclear.

In the present study, we investigated the effects of Alternol-induced oxidative stress on autophagy-associated signaling pathways in prostate cancer cells. Given the emerging role of p62 in redox regulation and proteostasis, we focused on determining whether ROS-mediated modification of p62 contributes to the cellular response to Alternol treatment. Our findings provide new insight into the relationship between oxidative stress, p62 regulation, and adaptive survival mechanisms in prostate cancer cells.

## 2. Materials and Methods

### 2.1. Cell Culture, Special Chemical Reagents, Plasmid Constructs, and Antibodies

Benign prostatic hyperplasia-1 (BPH1), human embryonic kidney (HEK)-293T, prostate cancer PC-3, DU145, 22RV1, and C4-2B cell lines were obtained from ATCC (Manassas, VA, USA) as described [11]. BPH1, PC-3, 22RV1, and C4-2B cells were cultured in RPMI 1640 media containing 10% fetal bovine serum and 1% penicillin/streptomycin in a 5% CO_2_ humidified atmosphere at 37 °C. HEK-293T and DU145 cells were cultured in DMEM media containing 10% fetal bovine serum and 1% penicillin/streptomycin in a 5% CO_2_ humidified atmosphere at 37 °C. Unless otherwise specified, cells were seeded overnight to allow attachment before treatment and were maintained at 60–80% confluency during experiments.

Alternol was obtained from Sungen Biosciences (Shantou, China). Chemicals of n-acetylcysteine (N-Ac), Chloroquine (CQ), STO-609, L-Arginine, PKC-ζ-inhibitor peptide, BATPA, Auranofin, and rapamycin were obtained from Cayman Chemicals (Arbor, MI, USA). The Luciferase assay system (E1501) and TMR-conjugated Halo ligand (G8251) were purchased from Promega (Madison, WI, USA). Lyso-Tracker Red (GC19882) was purchased from GlpBio (Montclair, CA, USA). Hydrogen peroxide (H_2_O_2_) and Annexin V-FITC Apoptosis Detection Kit were obtained from Sigma-Aldrich (St. Louis, MO, USA).

The pGL4.27_ARE/NRF2-SPE luciferase reporter construct [12] was a gift from Michael Ristow (Addgene #177775). The pMXs-puroGFP-p62/K7A-D69A (Addgene #38281), pMXs-puroGFP-p62 [13] (Addgene #38277), pMXs-GFP-LC3-RFP [14] (Addgene #117413), pCDH-EF1a-mCherry-EGFP-LC3B (Addgene #170446), and pMRX-IP-HaloTag7-LC3 (Addgene #184899) plasmids were kindly provided by Noboru Mizushima, who deposited them on Addgene (Watertown, Mass., USA). The plasmid constructs harboring the SQSTM1/p62WildType or Cys105/113A mutations were provided by Dr. Viktor Korolchuk (Newcastle University). The iScript cDNA Synthesis kit (#1708891) and iTaq SYBR Green Supermix (#1725121) were purchased from Bio-Rad (Hercules, CA, USA). Gentian Violet and TRIzol RNA isolation reagents were obtained from Thermo Fisher (Waltham, MA, USA).

SQSTM1/p62 (sc-28359), LKB1 (sc-32245), and β-Actin (sc-4778) were purchased from Santa Cruz Biotechnology (Santa Cruz, CA, USA). PARP (CST-9532), Caspase-3 (CST-9662), LC3β (CST-3868), AMPK (CST-5831), p-AMPK (T172) (CST-2535), p-AMPK(S485) (CST-2537), KEAP1 (CST-8047), Nrf2 (CST-12721), p-LKB1/S428 (CST-3482), phospho-SQSTM1/p62 (Ser403) (CST-39786), phospho-SQSTM1/p62 (Ser349) (CST-16177) and anti-Biotin (CST-5597) were purchased from Cell Signaling Technology (Danvers, MA, USA). p-LKB1/S307 (EMD:09478) was purchased from Merk Millipore (Burlington, MA, USA). anti-Halo (G9211) was purchased from Promega (Madison, WI, USA).

### 2.2. Western Blot, qPCR, and Protein Carbonylation Assay

Cells were seeded in 100 mm dishes or 6-well plates and allowed to attach overnight before treatment with the indicated compounds for the specified time periods. Cells were washed twice with cold PBS solution, then lysed with RIPA Buffer with phosphorylase and protease inhibitor (Cell Signaling Technologies, Danvers, MA, USA). Protein concentrations were determined with the Standard BCA Protein Assay Kit (Thermo Fisher, Waltham, MA, USA). The lysed samples were boiled and subjected to separation with 8% or 12% SDS-PAGE gel at 70–120 V. Proteins were transferred to the PVDF membrane. The membranes were blocked for 60 min with 5% no-fat milk solutions prepared in phosphate-buffered saline (PBS) with 0.1% Tween 20, incubated overnight at 4 °C with 1:1000 dilutions of the primary antibodies, and washed three times for 10 min each time with Tween 20 (1:1000 dilution) in PBS. Appropriate peroxidase-conjugated secondary antibody (1:5000 dilution) was used for 2 h at room temperature. Membranes were washed with Tween 20-PBS three times for 10 min. Protein bands were visualized using an ECL solution from Santa Cruz Biotech (Santa Cruz, CA, USA).

Total RNA samples were isolated using TRIzol RNA isolation reagents (Invitrogen). Gene expression was assessed using a SYBR Green-based quantitative RT-PCR assay. qPCR experiment and analysis were tested as described in our previous report [15]. PCR primer pair for the human *AOX1* is forward 5′-ATGCCTGTCTGATTCCCATCT-3′, reverse 5′-CATGACACTTGGCAATCCTCT-3′. The human 18S rRNA primer pair is forward 5′-CTACCACATCCAAGGAAGCA-3′ and reverse 5′-TTTTTCGTCACTACCTCCCCG-3′.

Protein carbonylation was evaluated using a biotin derivatization assay as described [16]. In brief, cells were seeded in 100 mm dishes and treated as indicated for the specified time periods prior to harvesting for protein extraction. The treated cells were harvested in cold PBS and cellular proteins were extracted under native conditions in G-lysis buffer (Guanidine HCl 6.0 M, Tris 50 mM, pH 8.3, EDTA 3.0 mM, Triton-X100 0.5% (*v*/*v*), and sodium iodoacetate 50 mM), as described [17]. Subsequently, the protein lysate was incubated in the dark with 5.0 mM biotin hydrazide for 2 h at room temperature. Biotin-conjugated proteins were reduced with 10.0 mM NaBH4 for 1 h. The excessively salty chemicals were removed with the Ultracel^®^-3K centrifugal filter (Merk Millipore, Darmstadt, Germany). The eluted proteins were loaded onto an SDS-PAGE gel, and carbonylated proteins on the PVDF membrane were detected using the HRP-linked anti-Biotin antibodies. Protein band density was acquired using NIH ImageJ software (version 1.54d; National Institutes of Health, Bethesda, MD, USA).

### 2.3. CRISPR/Cas9 Knockout (KO) System Stable Cell Line and Small Interfering RNAs

Cells were seeded in 6-well plates at approximately 50–60% confluency prior to transfection. *SQSTM1* knockout (KO) stable cell lines were established in C4-2B and PC-3 cells using a CRISPR plasmid system (sc-400099; Santa Cruz Biotechnology, Dallas, TX, USA). The manufacturer’s protocol was followed to generate the KO cell lines. Stable sublines expressing wild-type or Cys105/113A mutant p62 were subsequently established in C4-2B and PC-3 cells.

The small interfering RNAs (siRNAs) for the *SQSTM1* gene (a set of 4 ON-TARGETplus siRNAs) were purchased from Horizon Discovery Inc. A negative control siRNA was also included in the cell-based assays. Lipofectamine RNAiMAX transfection reagent was purchased from Thermo Fisher Scientific Inc. The transfection procedure was performed according to the manufacturer’s manual. siRNA transfection was performed for 48 h before subsequent treatments or analyses.

### 2.4. Flow Cytometry Analysis

PC-3 cells were cultured in a 6-well plate (1 × 10^5^/well) and then treated as indicated for 16 h. Apoptotic cell death was evaluated with flow cytometry as described in our previous publication [18]. Briefly, cells were harvested and washed with cold PBS, and then incubated with annexin V-binding buffer and 1 mg/mL propidium iodide for 10 min at room temperature according to the assay kit protocols (Thermo Fisher, Waltham, MA, USA). Flow cytometry data were analyzed by *FlowJo* software (version 10.8.1; BD Life Sciences, Ashland, OR, USA).

### 2.5. Electron Microscope (EM) Analysis

PC-3 cells were seeded onto 0.2% gelatin-coated 10 mm coverslips placed in 100 mm dishes at approximately 40–50% confluency and cultured overnight and then treated as indicated for 16 h. The cells were fixed with freshly prepared 2.5% glutaraldehyde in PBS at 4 °C for 3 h. And then washed twice with cold PBS. Dehydration was carried out sequentially in the dishes with methanol at different concentrations (20%, 40%, and 60%) for 5 min each, followed by an 80% methanol wash for 3 min and then a 100% methanol wash for 30 s, repeated five times. The coverslips with dishes were dried in vacuum-assisted desiccators overnight. The surface of the coverslip was sputter-coated in a vacuum with an electrically conductive 5 nm thick layer of gold-palladium alloy precision etching coating system. The EM images were recorded with a scanning electron microscope (FEI Quanta 200) at a lower voltage (∼1 kV) and low vacuum mode with a tilt of 30 °C [19].

### 2.6. Co-Immunoprecipitation

C4-2B or PC-3 cells were seeded in 145 mm dishes and cultured to approximately 80% confluency prior to the indicated treatments. Cells were washed twice with cold PBS on ice and then collected in CHAPS lysis buffer [40 mM HEPES pH7.4, 120 mM NaCl, 1 mM EDTA, 10 mM pyrophosphate, 10 mM glycerophosphate, 50 mM NaF, 1.5 mM Na3VO4, 0.3% (*w*/*v*) CHAPS], and a cocktail of protease inhibitors (1:1000, Sigma, St. Louis, MO, USA). The samples were sonicated for 20 seconds and then centrifuged at 12,000 rpm for 30 min at 4 °C. Supernatants were transferred to new tubes. Protein concentration was determined with a standard BCA protein assay kit (Thermo Fisher, Waltham, MA, USA). Cell lysates were incubated with 3 µg primary antibodies for 1 h before adding 30 μl of protein A/G agarose beads (sc-2003, Santa Cruz Biotechnology, Dallas, TX, USA). After incubation at 4 °C overnight, the samples were centrifuged at 5000 rpm for 3 min to pellet the beads. The beads were washed with CHAPS lysis buffer 3 times, followed by Western blot analysis.

### 2.7. Fluorescence Microscopy

Cells were seeded onto glass coverslips in 6-well plates at a density of 1 × 10^5^ cells/well and cultured overnight, and then transfected with the plasmids for 48 h. The cells were treated as indicated at different times. After washing with cold PBS three times, the cells were incubated with Hoechst 33342 dye (Thermo Fisher, Waltham, MA, USA) for 15 min at room temperature. Images were acquired with a Nikon Confocal Microscope (Nikon Instruments Inc., Melville, NY, USA).

### 2.8. Gentian Violet Assay

C4-2B and PC-3 cells were seeded in a 6-well plate (1 × 10^5^/well), grown overnight, and then treated with Alternol (0, 2.5, 5.0, 10 μM) for 24 h. After fixation in cold acetone, cells were stained with 0.2% gentian violet for 5 min. Cells were washed with distilled water for photos.

### 2.9. Previously Published Xenograft and RNA-Seq Dataset

Protein lysates extracted from previously published xenograft samples [20]. Transcriptomic data derived from Alternol-treated PC-3 cells were reported in our previous publication [10]. The dataset is publicly available through NCBI BioProject (PRJNA705723). Detailed experimental procedures regarding RNA extraction, sequencing, and ethical approval were described in that publication. In the present study, the previously published RNA-seq dataset was re-analyzed using Gene Set Enrichment Analysis (GSEA) to identify signaling pathways associated with oxidative stress, autophagy, and antioxidant responses. Data visualization was performed using the Xiantao Scholar bioinformatics platform.

### 2.10. Statistical Analysis

All quantitative data are presented as the mean ± SEM from at least three independent biological replicates. Representative images of non-quantitative data were selected from at least three independent experiments with similar results. Statistical significance was determined using Student’s *t*-test for comparisons between two groups. For multiple group comparisons, one-way or two-way analysis of variance (ANOVA) was performed, followed by Tukey’s post hoc test to determine specific differences between groups. All statistical analyses were conducted using SPSS software (Version 25.0, IBM Corp. Armonk, NY, USA). A *p*-value less than 0.05 was considered statistically significant. All experiments were independently repeated at least three times unless otherwise stated.

## 3. Results

### 3.1. Alternol Treatment Elicited Autophagy and Nrf2 Antioxidant Pathway in Prostate Cancer Cells

Our previous studies have shown that Alternol preferentially induces cell death in malignant cells through superoxide-dependent apoptotic mechanisms, accompanied by ER stress-associated immunogenic cell death (ICD) and impaired mitochondrial ATP production in xenograft tumor models [7,8,9,10,20,21,22,23].

**Figure 1:**
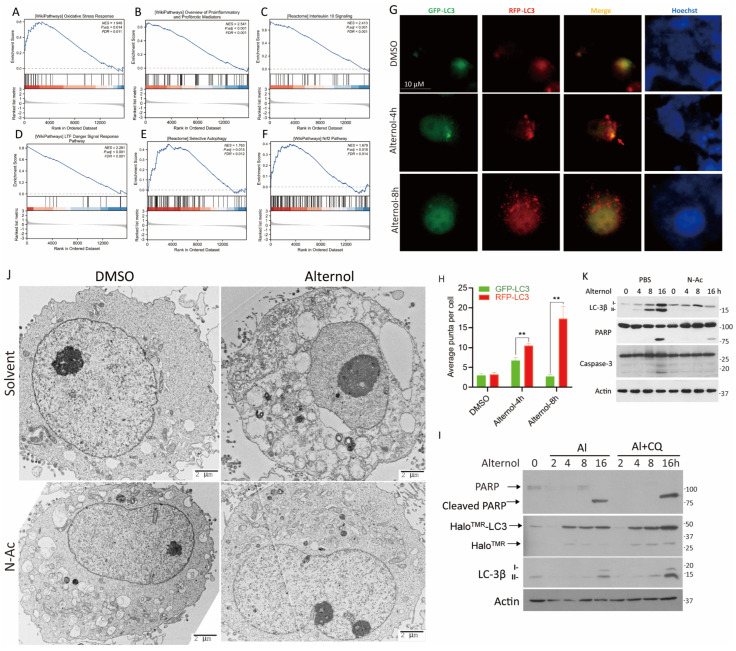
**Alternol treatment induces multiple transcriptomic alterations.** (**A**–**F**) GSEA was performed using a previously published RNA-seq dataset from the Alternol-treated PC-3 cells [10]. The visualization of the results was conducted at the web-based bioinformatic platform Xiantao Scholar (https://www.xiantaozi.com/, accessed on 12 June 2025). (**G**) PC-3 cells were transfected with the pMXs-GFP-LC3-RFP plasmid for 48 h and then treated with Alternol (10 μM) for 4–8 h. Fluorescent microscopic images were taken under a confocal microscope. (**H**) Quantitative data for the LC3 puncta per cell (LC3 dots) were summarized. Data are presented as mean ± SEM from three independent experiments (n = 3). Statistical significance was determined using one-way ANOVA followed by Tukey’s post hoc test. ** *p* < 0.01 versus control. (**I**) C4-2B cells were transfected with pMRX-IP-HaloTag7-LC3 plasmid for 48 h, incubated with TMR (100 μM) for 30 min, washed with PBS and then treated by Alternol (10 μM) with/without CQ (25 μM) for 0, 2, 4, 8, 16 h. Cells were harvested for Western blot analysis. (**J**) PC-3 cells were treated with Alternol (10 μM) with/without N-Ac (5 mM) for 6 h, followed by transmission electron microscopy. (**K**) PC-3 cells were treated with Alternol (10 μM) with/without N-Ac (5 mM) for 4, 8, 16 h. Cells were harvested for Western blot analysis with the indicated antibodies. An actin blot was used for protein loading control.

To further explore the molecular mechanisms associated with Alternol treatment, we re-analyzed our previously published RNA-seq dataset derived from the Alternol-treated PC-3 cell line [10]. A total of 101 upregulated and 66 downregulated genes were identified with a threshold of log_2_FC > 2 (Table S1). Gene set enrichment analysis (GSEA) suggested enrichment of oxidative stress response (Figure 1A), proinflammatory signaling (Figure 1B), IL10 signaling pathways (Figure 1C), and danger-associated signaling pathways (Figure 1D) following Alternol treatment. These findings are consistent with our previous reports showing that Alternol induces ICD through oxidative stress- and proinflammatory-mediated signaling in prostate cancer cells [9,10]. Notably, GSEA further identified enrichment of selective autophagy (Figure 1E) and the Nrf2-mediated antioxidant pathway (Figure 1F), which were selected for further mechanistic investigation.

To validate these transcriptomic findings, we verified the autophagy response in PC-3 cells using four complementary approaches. First, we employed the tandem fluorescent probe (pMXs/GFP-LC3-RFP reporter [24]), a standard marker for monitoring autophagy flux, to distinguish between early (GFP/RFP colocalization) and late (RFP-only) autophagic phases [25]. Following transient transfection and Alternol treatment for 4–8 h, we observed a significant accumulation of both GFP/RFP puncta at 4 h (Figure 1G), which is characteristic of an early autophagic response [25]. The subsequent increase in RFP-only puncta at 8 h provided clear evidence of an active autophagy flux in Alternol-treated cells [25]. Quantification of LC3 puncta is summarized in Figure 1H. To further assess the role of ROS in Alternol-induced autophagy, PC-3 cells expressing mCherry-EGFP-LC3B were treated with Alternol in the presence or absence of N-Ac. N-Ac largely abolished Alternol-induced accumulation of LC3-positive puncta (Figure S1A,B). Furthermore, GFP-p62 puncta co-localized with LysoTracker-positive acidic vesicular structures following Alternol treatment (Figure S1C,D), indicting the autolysosome formation.

Second, to further characterize autophagic flux, we employed the HaloTag-LC3 processing assay in C4-2B cells [26]. Following pulse-labeling with TMR-ligands, Alternol treatment significantly increased both Halo^TMR^–LC3 fusion protein and cleaved Halo^TMR^ products. This effect was further enhanced by CQ co-treatment (Figure 1I). Increased autophagy-associated signaling was accompanied by time-dependent PARP cleavage, which was further enhanced by CQ treatment (Figure 1I).

Thirdly, transmission electron microscopy (TEM) was performed to analyze the morphological autophagy features after Alternol treatment. TEM analysis revealed extensive cytoplasmic vacuolization following Alternol treatment, consistent with autophagic morphology (Figure 1J).

Fourth, we analyzed the processing of microtubule-associated protein 1A/1B-light chain-3 (LC3), a critical component in autophagy induction [25]. Alternol induced LC3 lipidation, as evidenced by increased LC3β-I to LC3β-II conversion in a ROS-dependent manner (Figure 1K). This autophagy response was accompanied by PARP cleavage and caspase-3 processing at the late time point (Figure 1K), consistent with our previous report [7]. Collectively, these data demonstrate that Alternol induces ROS-dependent autophagy coupled with apoptotic signaling in prostate cancer cells.

### 3.2. Alternol Treatment Was Associated with Activation of the LKB1-AMPK Pathway

**Figure 2:**
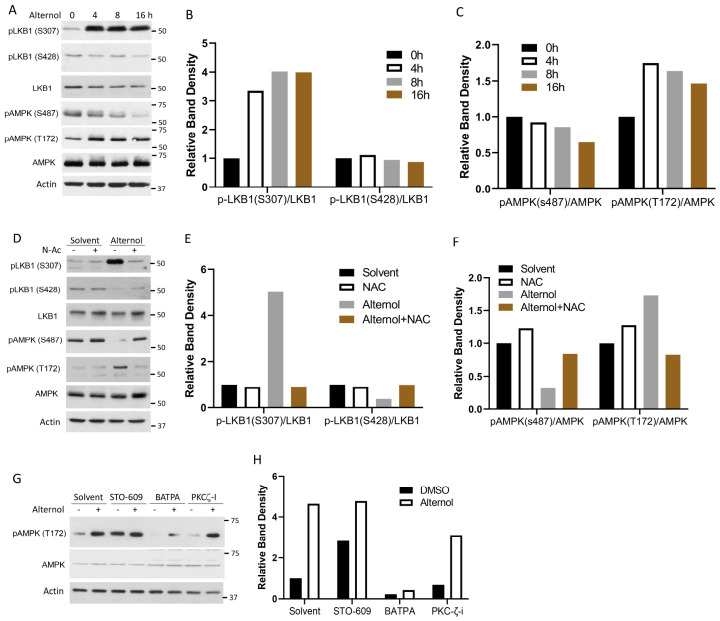
**Alternol treatment activated the LKB1/AMPK pathway.** (**A**) PC-3 cells were treated with Alternol (10 μM) at different times (0, 4, 8, 16 h). Protein band density data were generated using the NIH ImageJ software. (**B**,**C**) The relative values of protein band density were calculated after normalization against Actin bands. (**D**) PC-3 cells were treated with Alternol (10 μM) with/without N-Ac for 6 h, as indicated, followed by Western blot assay. (**E**,**F**) The relative values of protein band density were calculated after normalization against Actin blot bands. (**G**) PC-3 cells were treated as indicated for 4 h, followed by a Western blot assay. Note: Alternol (10 μM), STO-609 (10 μM), BATPA (10 μM), PKCζ-I (10 μM). (**H**) The relative values of pAMPK (T172) protein band density were calculated after normalization against AMPK and Actin bands. Data were normalized to the solvent control (set as 1).

**Figure 3:**
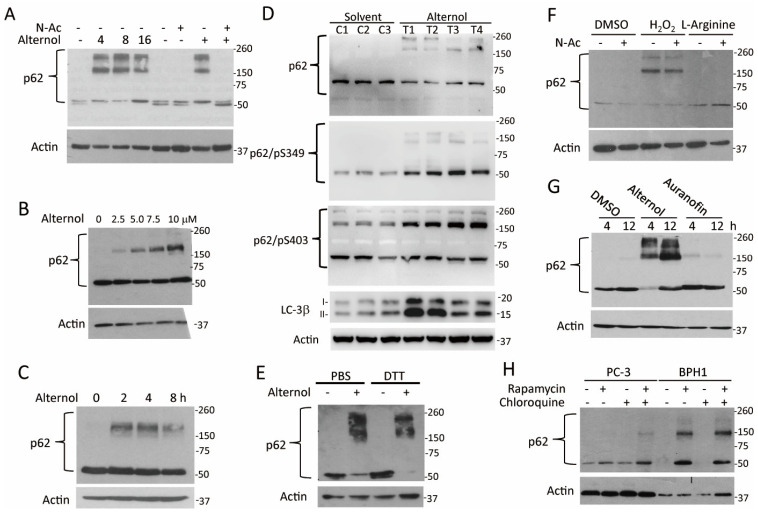
**Alternol induces p62 protein aggregation.** (**A**) PC-3 cells were treated with Alternol (10 μM) for different periods as indicated (the first half panel). PC-3 cells were pre-treated with N-Ac (5 mM) for 30 min, followed by Alternol (10 μM) for 4 h (the second half panel). (**B**) PC-3 cells were treated with Alternol at different concentrations as indicated for 4 h. (**C**) DU145 cells were treated with Alternol (10 μM) for up to 8 h and were harvested for Western blot assay. (**D**) Protein lysates extracted from previously published xenograft samples [20] of the solvent control group (C1–C3) and Alternol-treated group (T1–T4) were used for the Western blot assays. (**E**) PC-3 cells treated with or without Alternol (10 μM) for 4 h. The RIPA buffer-based cellular lysates were heated in PBS or DTT (100 mM) at 95 °C for 5 min before the Western blot assay. (**F**) PC-3 cells were pre-treated with N-Ac (5 mM) for 30 min, followed by the solvent, H_2_O_2_ (200 mM), or L-Arginine (0.5 mM) treatment for 6 h. (**G**) PC-3 cells were treated with the solvent DMSO, Alternol (10 μM), or Auranofin (5 μM) for 6 h. Whole-cell lysates were subjected to a Western blot assay. (**H**) PC-3 or BPH1 cells were treated with chloroquine (5 μM) or rapamycin (0.5 μM) as indicated for 6 h.

To investigate the upstream signaling mechanisms underlying Alternol-induced autophagy, we examined the LKB1–AMPK pathway, a central regulator of cellular energy stress responses [27,28,29]. Alternol treatment increased phosphorylation of LKB1 at the activating Ser307 site [30], while reducing phosphorylation at the inhibitory Ser428 site [31]. Consistent with alterations in LKB1 phosphorylation status, AMPK phosphorylation at Thr172 was also increased following Alternol treatment [32] (Figure 2A). In parallel, phosphorylation at the inhibitory Ser487 site of AMPK [33] was markedly reduced in a time-dependent manner (Figure 3A). Representative densitometric analysis indicated similar trends. Furthermore, pretreatment with N-Ac fully reversed all Alternol-induced alterations in LKB1 and AMPK phosphorylation (Figure 2D–F). Together, these results indicate that Alternol activates the LKB1–AMPK pathway in a ROS-dependent manner.

To determine if Ca^2+^/Calmodulin-dependent protein kinase (CaMKK) contributed to LKB1/AMPK pathway activation [34], we tested the effects of the CaMKK inhibitor, STO-609 [35], and the calcium-specific chelator, BAPTA [36], on Alternol-induced AMPK phosphorylation. Our results showed that STO-609 pretreatment largely reduced the basal level of AMPK Thr172 phosphorylation, it had no significant inhibitory effect on the Alternol-induced increase in AMPK Thr172 phosphorylation (Figure 2G,H). In contrast, calcium chelation by BAPTA markedly attenuated Alternol-induced AMPK phosphorylation. (Figure 2G). These data indicate that while CaMKK may be responsible for maintaining basal AMPK activity, cellular calcium signaling is specifically and critically involved in the Alternol-induced activation of AMPK Thr172 phosphorylation.

Previous studies have suggested that protein kinase C_ζ_ (PKC_ζ_) modulated LKB1-AMPK activation [37]. We assessed the effect of a myristylated PKC_ζ_ pseudo-substrate inhibitor [38] on Alternol-induced AMPK phosphorylation. As shown in Figure 2G, pretreatment with the PKC_ζ_ pseudo-substrate inhibitor had no obvious inhibitory effect on Alternol-induced AMPK phosphorylation at the Thr172 site, suggesting that PKC_ζ_ was not involved in Alternol-induced AMPK activation.

### 3.3. Alternol Induced p62 Protein Aggregation in Response to Oxidative Stress

p62 protein is a selective autophagy receptor that is typically degraded during autophagy activation [25,39]. We observed the accumulation of high-molecular-weight p62 species following Alternol treatment. These p62 species appeared as early as 4 h after Alternol treatment (Figure 3A). The formation of these high-molecular-weight p62 bands was abolished by N-Ac pretreatment, indicating an oxidative stress-mediated response, as previously reported [5]. Alternol induced p62 aggregation in a dose-dependent manner in both PC-3 and DU145 cells, as well as in previously published xenograft tumor samples [20] (Figure 3B,D). Notably, these high-molecular-weight bands were resistant to detergent and reducing agent DTT treatment (Figure 3E). Collectively, these findings support the accumulation of high-molecular-weight p62 species during Alternol-associated oxidative stress responses in vitro and in previously reported xenograft samples.

We next tested whether other ROS inducers could trigger similar p62 aggregation. Hydrogen peroxide (H_2_O_2_) was also associated with the accumulation of p62 aggregates, whereas the nitric oxide inducer L-arginine did not (Figure 3F). The inhibitor of redox-maintaining enzyme thioredoxin reductase (TrxR) Auranofin, a reported ROS-inducing agent [40,41], did not noticeably increase p62 aggregation under these conditions (Figure 3G). Furthermore, Rapamycin, a well-characterized mTOR inhibitor and autophagy inducer [42], did not noticeably induce p62 aggregation in malignant PC-3 cells, whereas increased high-molecular-weight p62 species were observed in benign BPH1 cells (Figure 3H), consistent with previous studies [5]. Combining Rapamycin with the autophagy blocker CQ resulted in only modest accumulation of p62 aggregates in both cell lines (Figure 3H). Collectively, these findings suggest that Alternol treatment is associated with accumulation of high-molecular-weight p62 species under oxidative stress conditions in malignant prostate cancer cells. The different responses between malignant and benign cells following Alternol or Rapamycin treatment deserve further mechanistic investigation.

### 3.4. p62 Protein Aggregation Was Driven by Protein Oxidation Rather than Phosphorylation, Ubiquitination, or Impaired Degradation

**Figure 4:**
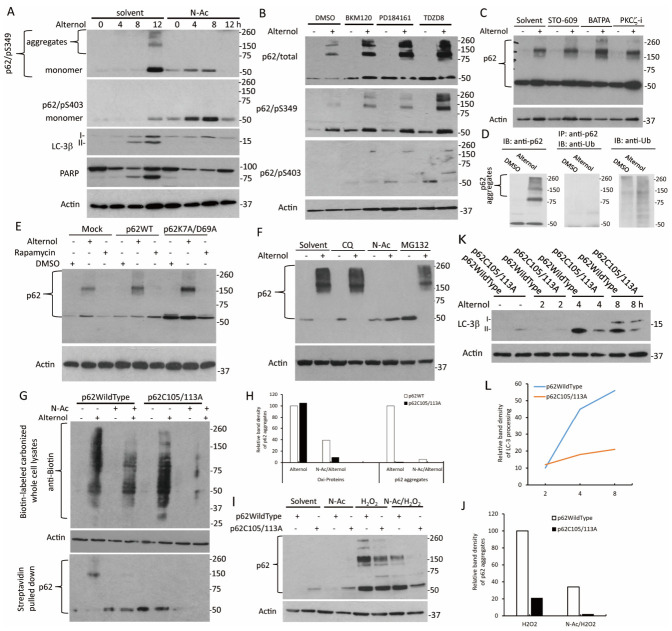
p62 protein aggregation is driven by protein oxidation rather than phosphorylation, ubiquitination, or impaired degradation. (**A**) PC-3 cells were pre-treated with N-Ac (5 mM) for 30 min, followed by Alternol (10 μM) for the periods as indicated. (**B**) PC-3 cells were pre-treated with the solvent DMSO, BKM120 (5 μM), PD184161 (10 μM), or TDZD8 (5 μM) for 30 min, followed by Alternol (10 μM) for 6 h. (**C**) PC-3 cells were pre-treated with the solvent DMSO, STO-609 (10 μM), BAPTA (20 μM), or PKCζ-i (5 μM) for 30 min, followed by Alternol (10 μM) for 6 h. (**D**) PC-3 cells were treated with the solvent DMSO or Alternol (10 μM) for 4 h. Equal amounts of cellular proteins were used for anti-p62 immunoprecipitation followed by anti-ubiquitin (anti-Ub) immunoblotting (**middle panel**). Whole cellular proteins were used for Western blot assays with the antibodies for p62 (**left panel**) or ubiquitin (**right panel**), as indicated. (**E**) PC-3 cells were transiently transfected with p62WT or p62K7A/D69A mutant constructs, as indicated. Mock transfection was conducted as the control. Transfected cells were treated with the solvent DMSO, Alternol (10 μM), or Rapamycin (0.25 μM) for 6 h. (**F**) PC-3 cells were pre-treated with the solvent DMSO, chloroquine (CQ, 5 mM), N-Ac (5 mM), or MG132 (10 μM) for 30 min, followed by Alternol treatment (10 μM) for 6 h. (**G**) HEK-293T cells were transiently transfected with the constructs as indicated. After treatment with N-Ac or Alternol alone or in combination, cells were subjected to protein carbonylation assay. The (**upper panel**): equal amounts of cellular proteins were subjected to anti-Biotin blotting, Actin blot served as the protein loading control; The (**lower panel**): equal amounts of proteins were subjected to streptavidin pulldown, followed by anti-p62 blotting. (**H**) The relative band density for oxidized proteins or p62 levels was calculated against the Alternol treatment samples (set a value of 100). (**I**) PC-3 cells stably transfected with p62 wild-type or Cys105/113A mutant constructs were treated with N-Ac, H_2_O_2_ (200 mM), or in combination for 4 h. (**J**) The relative band density for p62 aggregates was calculated against H_2_O_2_-treated p62/WT samples (set as value of 100). (**K**) PC-3 cells stably transfected with p62/WT or Cys105/113A mutant constructs were treated with the solvent DMSO or Alternol (10 μM) for the periods as indicated. (**L**) The relative band density for LC3β proteins were calculated against Alternol-treated p62/WT for 2 h samples (set as value of 10).

A previous report showed that p62 phosphorylation at the Ser403 site was linked to protein aggregation with polyubiquitinated proteins [43], while phosphorylation of p62 protein at Ser349 increased its affinity for the cargo KEAP1 protein [44,45]. We investigated whether p62 phosphorylation was involved in Alternol treatment-induced aggregation. As shown in Figure 4A, p62 protein monomers were strongly phosphorylated at both S349 and S403 sites at a very late time point (about 12 h) after Alternol treatment, which was completely different from the protein aggregation pattern starting at 4 h. In addition, p62 phosphorylation at S349 but not S403 was observed in the aggregates (Figure 4A). Similar results were also observed in PC-3 xenograft tissues (Figure 3D). Interestingly, N-Ac pre-treatment enhanced the phosphorylation at both sites on the monomers but abolished S349 phosphorylation on the aggregates (Figure 4A). These results indicated that while S349 phosphorylation occurs within the aggregates, these observations suggest that S349 phosphorylation may occur secondary to the aggregation process rather than initiating aggregate formation. The significance of enhanced p62 monomer phosphorylation at S349/S403 by N-Ac pretreatment is under further investigation.

To identify the signaling pathways potentially associated with Alternol-induced p62 phosphorylation, we tested a few pharmacological inhibitors for their effect on p62 aggregation and phosphorylation. As shown in Figure 4B, the pan-PI3K inhibitor BKM120 [46], pan-MAPK inhibitor PD184161 [47], and GSK-3 inhibitor TDZD8 [48] increased the levels of p62 protein aggregation and S349 phosphorylation, of which TDZD8 appeared to produce the greatest increase on both events. These inhibitors had only minimal effect on p62S403 phosphorylation (Figure 4B). These observations suggest that multiple kinase pathways may influence p62 phosphorylation status without being sufficient to account for the initiation of p62 aggregation.

We further examined if calcium-dependent signaling was involved in p62 protein aggregation based on our previous observations suggesting involvement of calcium signaling in Alternol-induced activation of the LKB1-AMPK pathway (Figure 4C). Our results showed that BATPA pre-treatment slightly enhanced Alternol-induced p62 protein aggregation, while CAMKK inhibitor STO-609 and PKCζ peptide inhibitor had no obvious effect on p62 aggregation.

To examine if p62 aggregation was due to polyubiquitination, we conducted a co-immunoprecipitation assay. Co-immunoprecipitation showed that Alternol-induced p62 aggregates did not contain high-molecular-weight ubiquitin bands (Figure 4D). It was reported that p62 protein forms self-oligomers via its Phox and Bem1p (PB1) domain during autophagy induction [49]. We then used a mutant p62 protein with a defect in oligomerization (p62/K7A.D69A) [13] and examined its effect on Alternol-induced p62 aggregation. After transient transfection overnight, Alternol treatment strongly induced p62 protein aggregates in p62K7A/D69A mutant-transfected cells compared to the p62 wild-type (p62/WT) transfected cells (Figure 4E). Rapamycin did not cause p62 aggregation (Figure 4E), similar to the previous results (Figure 3H). These data suggest that p62 protein oligomerization was not involved in Alternol-induced aggregation. Furthermore, blocking the autophagic (Chloroquine) or proteasomal (MG132) degradation machineries did not increase aggregate levels, confirming that the accumulation is not due to a defect in protein clearance (Figure 4F).

Given recent findings that p62 protein oxidation at cysteine-105/113 (Cys105/113) residues, leading to p62 aggregation and autophagy activation in the Drosophila system [5,6], we used a biotin derivatization-based protein carbonylation assay to assess cellular protein oxidation, as described in our recent publication [21] and from others [16]. The *SQSTM1* gene mutation on the Cys105/113A plasmid was used to examine the mutation’s effect on p62 protein oxidation and aggregation. Our results showed that Alternol treatment caused a dramatic accumulation of oxidized cellular proteins, which was largely reduced by N-Ac pre-treatment (Figure 4G(upper panel),H). These data were in line with our recent publication [21]. Crucially, the p62/Cys105/113A mutation abolished p62 aggregation even without NAC (Figure 4G(lower panel),H). These data were in line with the recent report for the involvement of p62 cystine-105/113 residues in protein oxidation and aggregation [5]. We observed similar results with H_2_O_2_ treatment, where the Cys105/113A mutation largely prevented aggregation (Figure 4I,J).

Lastly, we examined the functional impact of the Cys105/113A mutation on Alternol-induced autophagic response. While Alternol induced a robust, time-dependent increase in LC3 protein processing in wild-type cells, this response was significantly attenuated in cells overexpressing the Cys105/113A mutant (Figure 4K,L). These data collectively demonstrate that Alternol-induced p62 aggregation is mediated by direct protein oxidation at Cys105/113, a process essential for the subsequent activation of the autophagic response.

### 3.5. Oxidation of p62 Protein Facilitated KEAP1 Degradation and Nrf2 Activation

**Figure 5:**
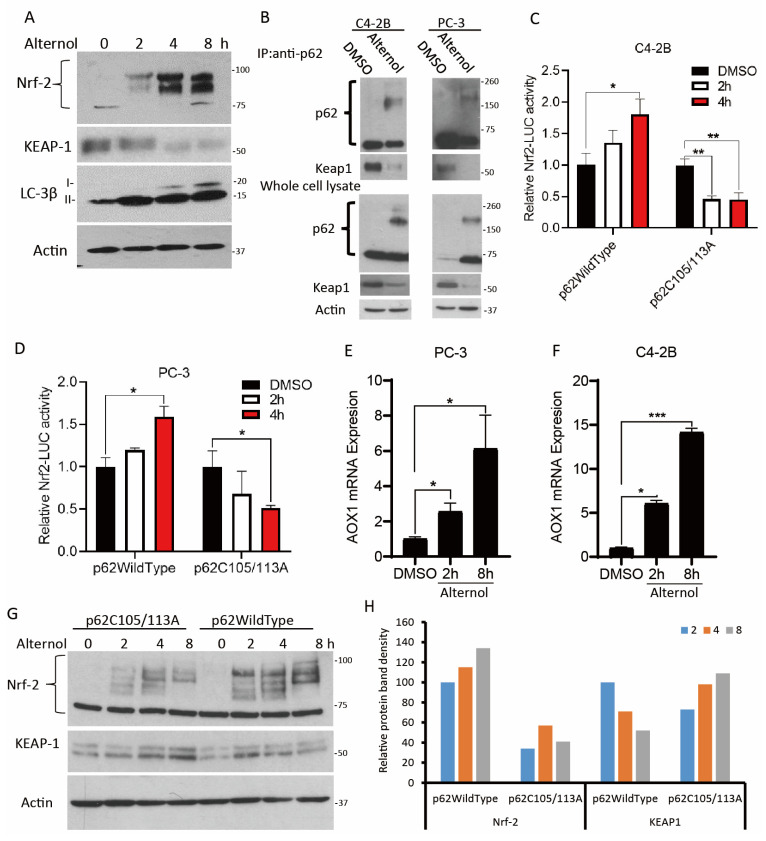
***SQSTM1* Cys105/133 mutation attenuates Nrf2 signaling.** (**A**) C4-2B cells were pre-treated with the solvent DMSO or Alternol (10 μM) for different periods, as indicated. (**B**) PC-3 or C4-2B cells were treated with the solvent or Alternol (10 μM) for 4 h, as indicated. The (**upper panel**): Equal amounts of cellular proteins were subjected to anti-p62 immunoprecipitation, followed by anti-KEAP1 Western blots; The (**lower panel**): whole cell lysates were subjected to regular Western blot assay. (**C**,**D**) C4-2B or PC-3 cells stably transfected with p62/WT or Cys 105/113A mutant constructs were transiently transfected with the Nrf2-LUC reporter plasmid for 48 h, followed by treatment with the solvent DMSO or Alternol (10 μM) for 2–4 h. Cells were harvested and lysed in a lysis buffer. Luciferase reporter activity was measured with the Luciferase Assay System (Promega E1501). Data are presented as mean ± SEM (n = 3). Data are presented as mean ± SEM from three independent experiments (n = 3). Statistical analysis was performed using one-way ANOVA followed by Tukey’s post hoc test. * *p* < 0.05, ** *p* < 0.01, *** *p* < 0.01 versus the indicated control group. The relative Nrf2-LUC reporter activity was calculated against the DMSO-treated p62/WT cells. (**E**,**F**) C4-2B or PC-3 cells were treated with the solvent DMSO or Alternol (10 μM) for 2–8 h. Cells were treated with Alternol (10 μM) for 2–8 h. Total cellular RNAs were extracted for real-time qPCR assays with the primers for the *AOX1* gene. (**G**) 22RV1 cells stably transfected with p62/WT or p62 Cys105/113A constructs, as indicated, were treated with Alternol (10 μM) for different periods (2, 4, 8 h). (**H**) The relative protein band density for Nrf2 or KEAP1 was calculated against the p62/WT 2 h treatment samples (set the value of 100).

p62 has been implicated in Nrf2-mediated antioxidant responses [3]. Since GSEA showed enrichment of the Nrf2 antioxidant pathway in Alternol-treated xenografts (Figure 1F), we verified the Nrf2 pathway activation induced by Alternol treatment. Our results demonstrated that Alternol treatment induced Nrf2 activation, accompanied by the appearance of high-molecular-weight Nrf2 complexes [50], and a reduction in KEAP1 protein levels (Figure 5A), possibly due to enhanced protein degradation [45]. Notably, these events synchronized with LC3 biosynthesis and processing, suggesting a functional link between Nrf2 activation and autophagy induction.

To investigate the involvement of p62 in KEAP1 degradation, we performed an anti-p62 co-immunoprecipitation (Co-IP) assay. Co-immunoprecipitation analysis showed that KEAP1 was associated with p62 under basal conditions; however, this interaction was markedly reduced following Alternol treatment (Figure 5B, the upper panels). This reduction occurred in parallel with p62 aggregation (Figure 5B, the lower panels). Subsequent Nrf2 transactivation was confirmed by an Nrf2-responsive luciferase reporter assay (Figure 5C,D) and further validated by the upregulation of the Nrf2 target gene *AOX1* (Figure 5E,F) [51,52]. Importantly, overexpression of the p62 Cys 105/113A mutant significantly reduced Nrf2 transcriptional activity compared with wild-type p62 (Figure 5C,D). Furthermore, overexpression of p62 Cys105/113A mutants also largely reduced Alternol-induced Nrf2 activation and was associated with sustained KEAP1 protein levels (Figure 5G,H). Collectively, these data suggest that p62 oxidation contributes to Alternol-induced activation of the KEAP1-Nrf2 signaling pathway under oxidative stress conditions.

### 3.6. Oxidation of p62 Protein Was Involved in Pro-Survival Autophagic Response

**Figure 6:**
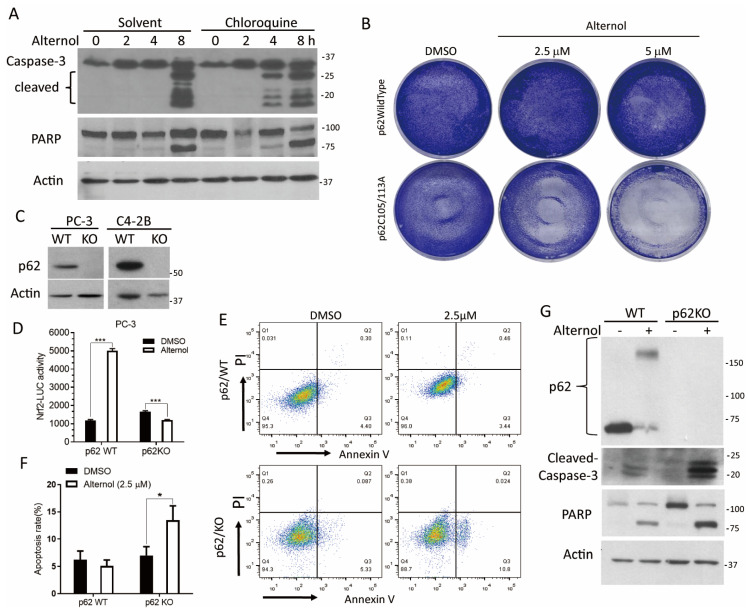
***SQSTM1* knockout abolishes Nrf2 activation and sensitizes Alternol-induced cell death.** (**A**) PC-3 cells were pre-treated with the solvent DMSO or chloroquine for 30 min, followed by Alternol (10 μM) for the periods, as indicated. (**B**) C4-2B cells stably transfected with p62/WT or p62 Cys105/113A construct were seeded in a 6-well plate, grown overnight, and then treated with Alternol (0, 2.5, 5.0 μM) for 24 h. After fixation, cells were stained with 0.2% gentian violet for 5 min and washed with double-distilled water 3 times. (**C**) PC-3 or C4-2B cells were transfected with CRISPR/Cas9 plasmids for gene editing. Stable clones with successful selection. *SQSTM1* gene knockout was confirmed in the Western blot assay. (**D**) PC-3 stable p62/KO or p62/WT subline cells were transiently transfected with the Nrf2-LUC reporter constructs for 48 h, followed by treatment with the solvent DMSO or Alternol (sublethal concentration at 5 μM) for 4 h. Cellular proteins were harvested for luciferase assays. Data are presented as mean ± SEM from three independent experiments (n = 3). *** *p* < 0.001 vs. Control, analyzed by one-way ANOVA followed by Tukey’s post hoc test.” (**E**) Stable PC-3 subline cells with p62/KO or p62/WT were treated with the solvent DMSO or Alternol (2.5 μM) for 16 h. Apoptotic cell death was assessed with an Annexin V/PI staining assay. Representative images were shown (panel (**E**)), and quantitative data were summarized in panel (**F**) Quantitative data from the Annexin-V/PI assay were summarized. Data are presented as mean ± SEM (n = 3). * *p* < 0.05 versus the indicated control group, analyzed by one-way ANOVA followed by Tukey’s post hoc test.” (**G**) The p62/WT and p62 KO subline C4-2B cells were treated with the solvent or Alternol (10 μM) for 16 h. Cellular proteins were subjected to Western blot assays with the antibodies as indicated.

To determine the functional role of Alternol-induced autophagy, we inhibited autophagic flux using chloroquine (CQ) in combination with Alternol treatment. Apoptotic cell death was assessed by caspase-3 activation and PARP cleavage [7]. Consistent with our previous report [7], Alternol induced caspase-3 activation and PARP cleavage at 8 h (Figure 6A). Notably, CQ co-treatment enhanced Alternol-induced apoptosis, with earlier activation of caspase-3 and PARP cleavage (Figure 6A). These results indicate that autophagy acts as a protective mechanism that delays Alternol-induced apoptotic cell death.

We next examined whether p62 oxidative modification influenced cellular sensitivity to Alternol treatment by overexpressing the p62-Cys105/113A mutant. Overexpression of the p62 Cys105/113A mutant significantly increased cellular sensitivity to a sublethal dose of Alternol compared with wild-type p62 (Figure 6B). These results suggest that p62 protein oxidation at these specific cysteine residues exerts a protective, pro-survival effect against Alternol-induced stress.

To further validate the role of p62, *SQSTM1* knockout (KO) cell lines were established in PC-3 and C4-2B cells (Figure 6C). As expected, loss of p62 markedly attenuated Alternol-induced Nrf2 transactivation (Figure 6D) and p62-deficient cells exhibited increased sensitivity to Alternol-induced cell death at sublethal concentrations (Figure 6E,F). This sensitization was accompanied by loss of p62 aggregation and enhanced caspase-3 activation and PARP cleavage in p62-KO cells (Figure 6G). Collectively, these data demonstrate that p62 oxidation at Cys 105/113 plays a key role in promoting cellular adaptation to Alternol-induced oxidative stress by supporting Nrf2 activation and autophagy, thereby contributing to cell survival.

## 4. Discussion

We previously demonstrated that Alternol induces profound oxidative stress and preferentially induces apoptotic cell death in cancer cells [7,9], accompanied by proinflammatory signaling and ICD [10]. In the present study, transcriptomic re-analysis of a previously published RNA-seq dataset further supported enrichment of inflammatory and oxidative stress-related pathways and additionally revealed enrichment of Nrf2-associated and autophagy-related signaling pathways. These results suggest a coordinated cellular response linking oxidative stress to adaptive survival mechanisms.

To further characterize this autophagy response, we utilized multiple experimental approaches in both cancer cell lines and xenograft models. A key finding of this study is the rapid and extensive aggregation of p62 following Alternol treatment. Although oxidation of p62 at Cys105 and Cys113 has been reported as an adaptive modification under aging-related oxidative conditions [5,6,53]. Our findings suggest that Alternol-induced oxidative stress promotes a more pronounced structural transition, leading to the formation of high-molecular-weight p62 aggregates. Notably, this process appears independent of ubiquitination, phosphorylation, or proteasomal inhibition and is resistant to reducing conditions, suggesting a distinct aggregation mechanism not previously described in this context.

In addition, using a HaloTag-LC3 flux assay, we further examined the functional consequence of this aggregation. Alternol induced robust autophagic activation, as reflected by increased LC3-II levels and enhanced autophagic flux. However, sustained accumulation of autophagy-related products, together with processing of HaloTag substrates, suggests that autophagic capacity eventually becomes saturated. This indicates a transition from efficient adaptive clearance to proteostatic stress, in which excessive cargo load exceeds degradative capacity, leading to cellular dysfunction.

Importantly, our findings suggest that p62 aggregation may participate in adaptive survival-associated responses during early Alternol-induced stress. We show that aggregated p62 facilitates KEAP1 sequestration, thereby promoting rapid activation of the Nrf2 antioxidant pathway. Disruption of p62 oxidation using the Cys105/113A mutant significantly impaired aggregate formation, attenuated Nrf2 activation, and increased sensitivity to Alternol-induced cell death. These findings support a model in which p62 oxidation-dependent aggregation functions as a redox-responsive platform that enables cancer cells to mount antioxidant defenses under therapeutic stress. However, when this adaptive response is overwhelmed, persistent proteostatic stress contributes to apoptotic cell death. However, it is not clear mechanistically how it occurred for the accumulation of the oxidized p62 protein aggregates after Alternol treatment. It is well known that p62 protein would be degraded together with its ubiquitinated cargo proteins inside the autolysosome body during autophagy flux [27,54]. In addition, further investigation is also necessary to determine if the oxidized p62 protein aggregates are resistant to lysosomal digestion or Alternol treatment enhances SQSTM1 gene expression, leading to p62 protein accumulation over autophagic degradation.

Furthermore, we observed a temporal dissociation between p62 oxidation/aggregation and its phosphorylation status. Although p62 phosphorylation at Ser349 and Ser403 has been implicated in Nrf2 activation and selective autophagy [44,55,56,57]. We found that Ser349 phosphorylation occurred at later time points, following the peak of Nrf2 activation. Interestingly, ROS scavenging enhanced rather than suppressed S349 phosphorylation of monomeric p62, suggesting that this modification may be regulated independently of early oxidative signaling and may represent a secondary regulatory event. The involvement of kinases such as TBK1 or CK2, as well as phosphatase-mediated regulation under oxidative stress, requires further investigation [43,58,59].

In conclusion, our study defines a functional “oxidation-aggregation” axis of p62 that regulates cellular adaptation to Alternol-induced oxidative stress. Genetic depletion of p62 or disruption of its oxidation sites markedly sensitizes prostate cancer cells to Alternol, highlighting the importance of this pathway in maintaining cellular resilience [39,45,60,61,62,63,64]. We propose that oxidized p62 aggregates serve dual functions: as signaling hubs that promote Nrf2-dependent survival responses, and as proteostatic burdens that contribute to eventual cellular collapse when excessive. Although the precise molecular mechanism by which p62 aggregates regulate KEAP1 turnover remains to be clarified, our findings provide a mechanistic framework for targeting redox-regulated proteostasis in cancer therapy.

## 5. Conclusions

In summary, this study identified Alternol as a potent inducer of a coordinated p62-Nrf2-autophagy survival axis in prostate cancer cells. The centerpiece of our findings is the discovery that Alternol-induced oxidative stress at Cys105/113 drives a rapid, large-scale aggregation of p62, a structural transition that serves as a vital signaling scaffold for KEAP1 degradation. Unlike previously reported oxidation events, this p62 aggregation occurs independently of phosphorylation and serves as a primary “redox sensor” to activate antioxidant defenses. Our results demonstrate that genetic depletion of p62 or disruption of its aggregation capacity (Cys105/113) significantly sensitizes cancer cells to Alternol, shifting the cellular balance from adaptive survival toward apoptosis. These insights underscore p62 protein aggregation as a critical determinant of cancer cell resilience and a promising target for enhancing pro-oxidant therapeutic strategies.

## Data Availability

The RNA-seq dataset analyzed in this study is publicly available in the NCBI BioProject database under accession number PRJNA705723.

## Supplementary Materials

The following supporting information can be downloaded at https://www.mdpi.com/article/10.3390/antiox15060779/s1, Figure S1: Alternol induces ROS-dependent accumulation of LC3-positive autophagic vesicles. (A) PC-3 cells transiently expressing the tandem fluorescent reporter mCherry-EGFP-LC3B were treated with DMSO (solvent), Alternol (10 μM), N-acetylcysteine (N-Ac, 5 mM), or N-Ac plus Alternol for 4 h. Representative fluorescence images showing EGFP-LC3 (green), mCherry-LC3 (red), merged images, and DAPI nuclear staining (blue) are presented. Alternol treatment induced accumulation of GFP/mCherry double-positive puncta, which was largely abolished by N-Ac pretreatment. (B) PC-3 cells expressing mCherry-EGFP-LC3B were treated as indicated for 16 h. Persistent accumulation of LC3-positive puncta was observed following Alternol treatment, whereas N-Ac prevented puncta formation. (C) PC-3 cells transiently expressing GFP-p62 were treated with DMSO or Alternol (10 μM) and stained with LysoTracker Red for 30 min. Representative images of GFP-p62 (green), LysoTracker (red), Hoechst (blue), and merged images are shown. Partial co-localization of GFP-p62-positive puncta with LysoTracker-positive acidic vesicular compartments was observed following Alternol treatment. (D) Quantification of GFP-p62, LysoTracker, and GFP-p62 puncta overlapping with LysoTracker-positive vesicles was performed using ImageJ. At least 30 cells were evaluated for each condition. The average number of puncta per cell was normalized to the average GFP-p62 puncta number in the solvent control group (set as 1). Data are presented as mean ± SEM from three independent experiments (n = 3). Table S1: RNA sequencing analysis results of Alternol-treated prostate cancer PC-3 cells.

## Abbreviations

The following abbreviations are used in this manuscript:

SQSTM1/p62	Sequestosome 1
Nrf2	Nuclear factor erythroid 2-related factor 2
KEAP1	Kelch-like ECH-associated protein 1
LC3	Microtubule-associated protein 1A/1B-light chain 3
AOX1	Aldehyde oxidase 1
ROS	Reactive oxygen species
NAC	N-acetylcysteine
CQ	Chloroquine
ICD	Immunogenic cell death
ER stress	Endoplasmic reticulum stress
GSEA	Gene set enrichment analysis
TEM	Transmission electron microscopy
Co-IP	Co-immunoprecipitation
qPCR	Quantitative real-time polymerase chain reaction
SDS-PAGE	Sodium dodecyl sulfate-polyacrylamide gel electrophoresis
DTT	Dithiothreitol
HMW	High-molecular-weight
LKB1	Liver kinase B1
AMPK	AMP-activated protein kinase
CHIP-seq	Chromatin immunoprecipitation sequencing
WT	Wild-type
KO	Knockout
