# Supplementary material for: Alternol-Induced Oxidative Modification of SQSTM1/p62 Is Associated with Nrf2 Signaling and Autophagy-Related Responses in Prostate Cancer Cells
Source: Antioxidants (Basel). 2026 Jun 22;15(6):779. doi: 10.3390/antiox15060779 (PMC13295585; doi:10.3390/antiox15060779)
Supplement: Supplementary file 1 [file antioxidants-15-00779-s001.zip › Figure S1. Alternol induces ROS-dependent accumulation of LC3-positive autophagic vesicles..pdf]

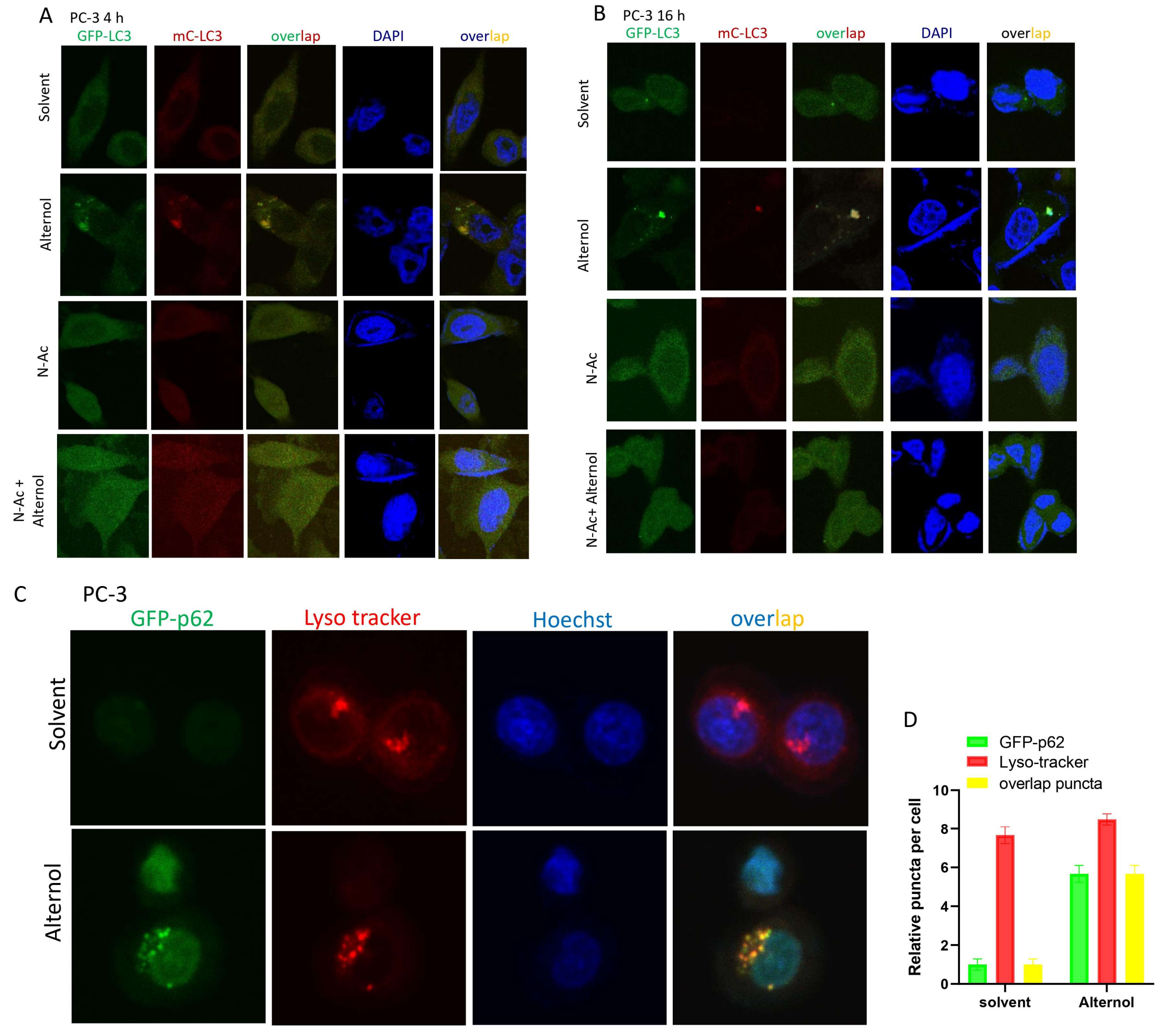

Figure S1. Alternol induces ROS-dependent accumulation of LC3-positive autophagic vesicles. (A) PC-3 cells transiently expressing the tandem fluorescent reporter mCherry-EGFP-LC3B were treated with DMSO (solvent), Alternol (10 μM), N-acetylcysteine (N-Ac, 5 mM), or N-Ac plus Alternol for 4 h. Representative fluorescence images showing EGFP-LC3 (green), mCherry-LC3 (red), merged images, and DAPI nuclear staining (blue) are presented. Alternol treatment induced accumulation of GFP/mCherry double-positive puncta, which was largely abolished by N-Ac pretreatment. (B) PC-3 cells expressing mCherry-EGFP-LC3B were treated as indicated for 16 h. Persistent accumulation of LC3-positive puncta was observed following Alternol treatment, whereas N-Ac prevented puncta formation. (C) PC-3 cells transiently expressing GFP-p62 were treated with DMSO or Alternol (10 μM) and stained with LysoTracker Red for 30 min. Representative images of GFP-p62 (green), LysoTracker (red), Hoechst (blue), and merged images are shown. Partial co-localization of GFP-p62-positive puncta with LysoTracker-positive acidic vesicular compartments was observed following Alternol treatment. (D) Quantification of GFP-p62, LysoTracker, and GFP-p62 puncta overlapping with LysoTracker-positive vesicles was performed using ImageJ. At least 30 cells were evaluated for each condition. The average number of puncta per cell was normalized to the average GFP-p62 puncta number in the solvent control group (set as 1). Data are presented as mean ± SEM from three independent experiments (n = 3).
